# Fabrication and Application of Dual-Modality Polymer Nanoparticles Based on an Aggregation-Induced Emission-Active Fluorescent Molecule and Magnetic Fe_3_O_4_

**DOI:** 10.3390/polym11020220

**Published:** 2019-01-28

**Authors:** Lingyun Wang, Meiying Huang, Hao Tang, Derong Cao, Yu Zhao

**Affiliations:** 1 School of Chemistry and Chemical Engineering, South China University of Technology, Guangzhou 510640, China; hmy_1015@126.com (M.H.); haotang@scut.edu.cn (H.T.); drcao@scut.edu.cn (D.C.); 2 Shanghai Key Laboratory of Magnetic Resonance and Department of Physics, East China Normal University, 3663 North Zhongshan Road, Shanghai 200062, China; zhaoyu2013022063@163.com

**Keywords:** dual-modality, AIE, MRI, bioimaging

## Abstract

Fluorescent magnetic nanoparticles (NPs) utilized for imaging hold great promise for biomedical applications, but it remains a challenging task. Here, we report novel dual-modality NPs using an aggregation-induced emission (AIE)-active and near-infrared (NIR) emissive dye (TPAS) and magnetic Fe_3_O_4_ as the core, and biocompatible polymer Pluronic F-127 as the encapsulation matrix by self-assembly procedures. The obtained fluorescent-magnetic AIE NPs have both high fluorescence quantum yield (13.8%) at 700 nm and high magnetic saturation value. With good photostability and biocompatibility, the resulting NPs show effective MRI ability, but also a stain in cytoplasm with a strong NIR fluorescent signal.

## 1. Introduction

As a noninvasive imaging technology, magnetic resonance imaging (MRI) possesses high spatial resolution and desired tissue penetration depth, but it suffers from low sensitivity and resolution [1,2]. On the other hand, fluorescence imaging has high sensitivity but fails to provide quantitative evaluation [3]. So, the integration of MRI and fluorescence imaging into a single probe will afford multimodal probe with synergistic benefits, which is highly desirable in bioimaging and diagnosis and provide more effective and accurate information about physiological functions [4,5,6,7,8,9,10].

The development of multimodal imaging is dependent on individual imaging probes [11]. Superparamagnetic iron (III) oxide particles are often chosen as multimodal imaging components [12,13] because of their strong MIR signals and enhancement of local contrast of magnetic resonance imaging. Meanwhile, aggregation-induced emission (AIE)-active fluorophores has opened a venue with great potential for high resolution imaging [14]. Upon encapsulation by polymer matrices, the resulting AIE dots become much brighter instead of undergoing weakened or quenched fluorescence [15]. In addition to the improved photostability, excellent colloidal stability in aqueous media and biological buffers, AIE dots are highly promising candidates as fluorescent trackers in vivo [16,17,18,19,20,21,22]. Moreover, near-infrared (NIR; 650–900nm) fluorescent probes have attracted intense interest due to less damage to living cells, better tissue penetration, improved the image sensitivity and lower interference from background auto-fluorescence. Considering the great significances of both AIE and NIR-emission, developing facile fabrication and application of dual-modality nanoparticles based on an AIE-active NIR fluorescent molecule and magnetic Fe_3_O_4_ is urgent and promising. But it remains a challenging task, because Fe_3_O_4_ nanoparticles are also excellent quenchers for most dyes and the synthesis of AIE-active NIR fluorescent molecule is time-consuming and tedious [19].

Herein, we have designed a novel nanoplatform for multimodal imaging. Amphiphilic surfactant (Pluronic F-127) provide the environment for the aggregation and self-assembly of hydrophobic substances. The NIR-emissive AIEgen and Fe_3_O_4_ NPs were encapsulated with Pluronic F-127, resulting in AIE–Fe hybrid nanodots, which exhibit stable and bright NIR emission under one-photon with excellent colloidal stability in biological environments. In vitro experiments show that AIE–Fe hybrid nanodots with high NIR fluorescence efficiency and magnetic susceptibility have been achieved, which is promising to facilitate their biological applications in multimodal imaging.

## 2. Materials and Methods

### 2.1. Chemicals and Instruments

All reagents and starting materials are commercially available and were used without further purification, unless otherwise noted. 4-Bromo-*N*,*N*-di-*para*-tolylaniline was purchased from Aladdin (Shanghai, China). Bis(hexyleneglycolato)diboron, Pd(Pph_3_)_4_, Pd(dppd)Cl_2_ were purchased from J&K Scientific Ltd (Beijing China). Pluronic F-127 and3-(4,5-dimethyl-2-thiazolyl)-2,5-diphenyl-2-H-tetrazolium bromide (MTT) were purchased from Sigma-Aldrich (St. Louis, MO, USA). Deionized water (18.2 MΩ cm resistivity) from a Milli-Q water system (Millipore, Bedford, MA, USA) was used throughout the experiments before being used as solvents. All other reagents and solvents were of analytical grade and used without further purification. Nuclear magnetic resonance spectra were recorded on Bruker Avance III 400 MHz (Bruker, Bremen, Germany) and chemical shifts are expressed in ppm using TMS (tetramethyl silane) as an internal standard. The UV–vis absorption spectra were recorded using a Helios Alpha UV–vis scanning spectrophotometer (Thermo Scientific, Bremen, Germany). Fluorescence spectra were obtained with a Hitachi F-4500 FL spectrophotometer (Tokyo, Japan) with quartz cuvette (path length = 1 cm). Solid state PL efficiencies were measured using an integrating sphere (C-701, Labsphere Inc.) with a 365 nm Ocean Optics LLS- Light Emitting Diode as the excitation source, and the laser was introduced into the sphere through the optical fiber. Dynamic Light Scattering (DLS) and zeta potential measurement was performed using a Malvern Zetasizer Nano ZS size analyzer (Malvern, Herrenberg, Germany) at room temperature. Transmission electron microscopy (TEM) images were obtained using a transmission electron microscope (TEM, JEM-2100F, Tokyo, Japan). The cellular imaging was performed on an Olympus IX71 microscope (Olympus, Tokyo, Japan) with mercury lamp as the excitation source.

The quantum yields of AIE–Fe nanodots were measured on the Hamamatsu absolute PL quantum yield spectrometer Quantaurus-QY C11347 (Hamamatsu Photonics, Hamamatsu, Japan) equipped with excitation light source of xenon lamp, monochromater and emission light collector of an integration sphere. The detector is a back-thinned charge-coupled device (CCD) sensor with high measurement sensitivity. Fe_3_O_4_ nanoparticles were purchased from Nanjing Nanoeast Biological Technology Co. Ltd (Nanjing, China).

### 2.2. Synthesis of Target Dye (TPAS)

#### 2.2.1. Synthesis of 1a

4-Bromo-*N*,*N*-di-p-tolylaniline (500.0 mg, 1.4 mmol), bis(pinacolato)diboron(431.7 mg, 1.7 mmol) and potassium acetate (412.2 mg, 4.2 mmol) were dissolved in 50 mL dioxane and added to a 100 mL flask. Under the nitrogen atmosphere, 5 mol% Pd(dppf)Cl_2_ (51.2 mg, 0.07 mmol) was added to the flask quickly and the mixture was heated to 100 °C. After reflux for 16 h, the mixture was concentrated using the rotary evaporators and purified by silica gel column chromatography with the eluent of petroleum ether-ethyl acetate (20:1, *v*/*v*). The white powder product with a yield of 88 % was obtained. ^1^H NMR (400 MHz, CDCl_3_, δ): 7.67 (d, 2H, J = 7.6 Hz), 7.11 (d, 4H, J = 7.9 Hz), 7.04 (d, 4H, J = 7.7 Hz), 7.01 (d, 2H, J = 7.7 Hz), 2.35 (s, 6H), 1.37 (s, 12H).

#### 2.2.2. Synthesis of 1b

1a (280.0 mg, 0.7 mmol) and 5-bromo-2-thiophenecarbaldehyde (200.0 mg, 1.05 mmol) were dissolved in 40 mL THF. Upon the nitrogen atmosphere, 3.5 mL 2 M potassium carbonate and 40 mg (5 mol%) Pd(PPh_3_)_4_ were added into the solution successively. After 24 h reaction, the mixture was poured into 100 mL water and extracted with dichloromethane until the aqueous phase became colorless. The organic phase was dried with NaSO_4_ and concentrated by the rotary evaporators and purified by silica gel column chromatography with the eluent of petroleum ether- ethyl acetate (10:1, *v*/*v*). The product is yellow solid with a yield of 60 %. ^1^H NMR (400 MHz, CDCl_3_, δ,): 9.77 (s, 1H), 7.62 (d, 1H, J = 3.9 Hz), 7.41 (d, 2H, J = 8.7 Hz), 7.20 (d, 1H, J = 4.4 Hz), 7.04 (d, 4H, J = 8.4 Hz), 6.96 (d, 4H, J = 8.4 Hz), 6.92 (d, 2H, J = 8.7 Hz), 2.26 (s, 6H).

#### 2.2.3. Synthesis of TPAS

1b (50.0 mg, 0.13 mmol) and malononitrile (17.0 mg, 0.26 mmol) dissolved in 20 mL EtOH were added into a 50 mL flask. Under the nitrogen atmosphere, two drops of triethylamine were dropped into the mixture and heated to 80 °C for 6 h. Next, red solution was concentrated by the rotary evaporators and purified by silica gel column chromatography with the eluent of hexane - ethyl acetate (5:1, *v/v*) to obtain the red solid with a yield of 27 %. ^1^H NMR(400 MHz, CDCl_3_, δ): 7.73 (s, 1H), 7.67 (d, 1H, J = 4.2 Hz), 7.49 (d, 2H, J = 9.0 Hz), 7.30 (d, 1H, J = 4.2 Hz), 7.13 (d, 4H, J = 8.2 Hz), 7.05 (d, 4H, J = 8.5 Hz), 6.98 (d, 2H, J = 8.9 Hz) 2.35 (s, 6H). ^13^C NMR (100 MHz, CDCl_3_, δ): 157.69, 150.42, 144.18, 140.55, 134.39, 133.00, 130.37, 127.66, 125.85, 124.09, 123.16, 120.62, 114.81, 113.94, 74.83, 21.06. HRMS (ESI, *m/z*), [M + H]^+^ calcd for C_28_H_22_N_3_S 432.1529; found, 432.1520.

### 2.3. Fabrication of AIE–Fe Hybrid Nanodots

Firstly, 0.5 mg TPAS, 10 mg Pluronic F127 and 0.5 mg oleic acid modificating Fe_3_O_4_ were dissolved in 1-mL tetrahydrofuran (THF), and then injected the above solution into 10 mL deionized water under the ultrasonic condition. After 20 min, the THF was removed through bubbling N_2_ gas and heating at 60 °C. The resulting mixture was filtrated with 0.22-μm filter and filtrate was stored in a refrigerator at 5 °C.

### 2.4. In Vitro Cytotoxicity Assay

HeLa cells used in this study were purchased from Cobioer Biosciences Co., Ltd. (Nanjing, China). To determine the cytotoxicity of AIE–Fe nanodots, methyl thiazolyl tetrazolium (MTT, Sigma Aldrich, St. Louis, MO, USA) assays were performed. Briefly, HeLa cells were seeded in 96-well plates and cultured in a CO_2_ incubator for 12 h at 37 °C. Then, the old medium was replaced with fresh medium containing various concentrations of AIE–Fe nanodots (5–50 ppm). The cells were incubated in the CO_2_ incubator for 24 h. Subsequently, MTT was added to each well for 4 h at 37 °C. Then, DMSO was added to each well, and each plate was agitated on a plate shaker for 10 min. The absorbance was measured at 570 nm using a microplate reader.

### 2.5. Fluorescence Imaging in HeLa Cells with AIE–Fe Nanodots

The HeLa cell lines were cultured in dulbecco’s modified eagle medium (DMEM) medium supplemented with 10% (*v/v*) calf serum, penicillin (100 U/mL), and streptomycin (100 mg/mL). The cells were seeded in laser confocal fluorescence microscope (LCFM) culture dishes and maintained at 37 °C in a humidified atmosphere containing 5% CO_2_. When the whole cells took up 60–70% space of culture dishes, the cells were further incubated with AIE–Fe nanodots (10 ppm) for 30 min at 37 °C. The cellular imaging was performed on an Olympus IX71 microscope with mercury lamp as the excitation source.

### 2.6. In Vitro MRI Studies

In vitro MRI studies were conducted on a 7Tesla MRI Bruker ClinScan using a 72-mm volume coil. The longitudinal relaxation time (T1) of the AIE–Fe nanodots in aqueous solutions with different concentrations of Fe_3_O_4_ were measured using an inversion recovery spin echo sequence.

## 3. Results and Discussion

### Fabrication of Fe_3_O_4_@TPAS Dots

**Figure 1:**
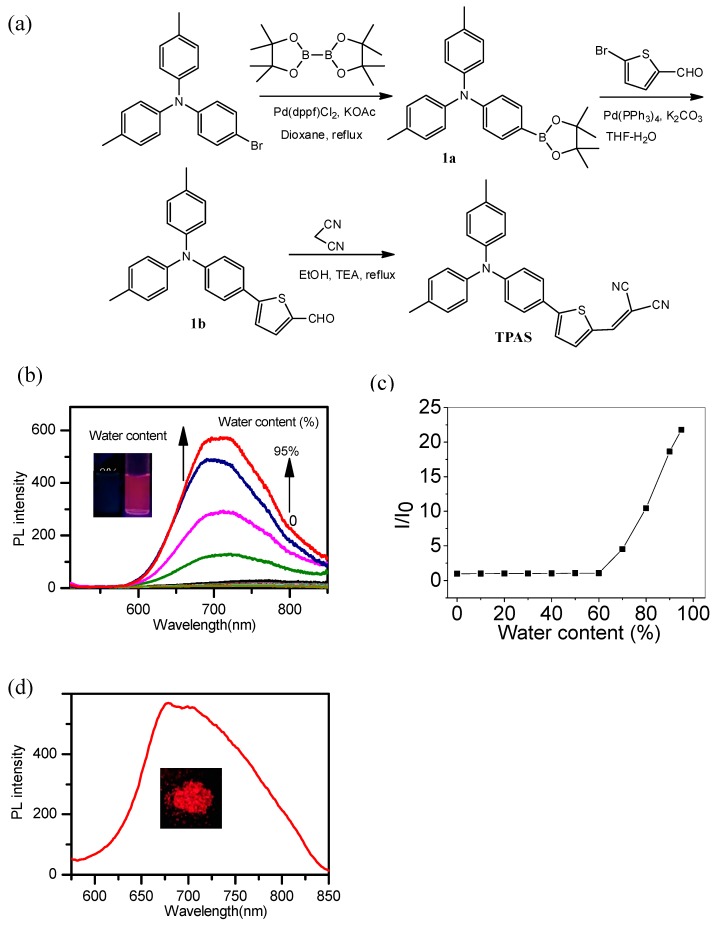
(**a**) Synthetic routes of target dye TPAS. (**b**) The emission spectra of TPAS (10 μM) in CH_3_CN/water mixtures with different fraction of water (λ_em_ = 494 nm). (**c**) The fluorescence intensity ratio in presence of different fraction of water. (**d**) TPAS in solid state. Inset: photographs of TPAS in solid state under 365 nm UV irradiation.

As shown in Figure 1a, NIR fluorophore (namely, TPAS, due to presence of triphenylamine section) was synthesized. Firstly, the bromic group of 4-bromo-*N*,*N*-di-*para*-tolylaniline was substituted by boric acid ester to yield 1a. The following Suzuki coupling reaction between 1a and 5-bromothiophene-2-carbaldehyde to generate 1b. Finally, TPAS was obtained by Knoevenagel condensation reaction between 1b and malononitrile. The chemical structures of intermediates and TPAS are characterized by NMR and HRMS (Figures S1–S5, Supporting information).

The cyan moiety is highly electron-deficient, so it can play the role of the electron-acceptor (A), and the triphenylamine can be the electron-donor (D). The D–A structure endows TPAS with a large dipole and facilitate to emission at long wavelength. The AIE property of TPAS was explored in CH_3_CN-water mixture. As showed in Figure 1a, TPAS in CH_3_CN was non-emissive and showed no obvious fluorescence enhancement when the water fraction (*f_w_*) was below 70%. Afterwards, NIR emission centered at 700 nm intensified swiftly. The highest emission enhancement was recorded with *f_w_* of 95%, which is 22-fold higher than that in CH_3_CN solution. In addition, TPAS showed the strong solid-state emission peak at 680 nm (Figure 1b). So, TPAS features the unique AIE characteristics.

**Figure 2:**
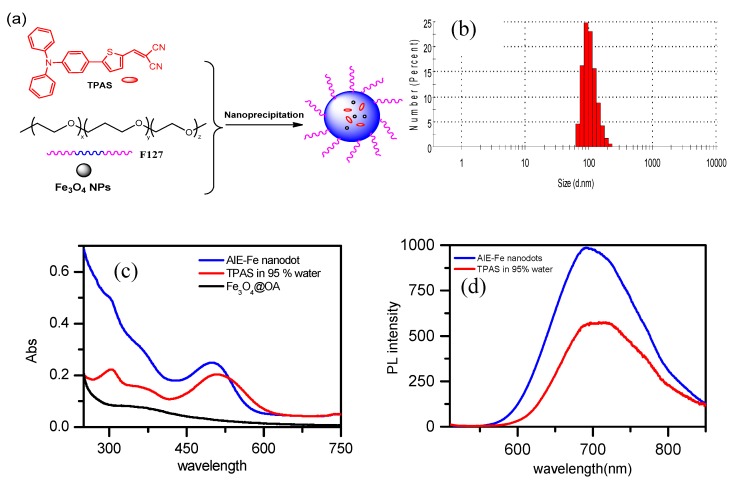

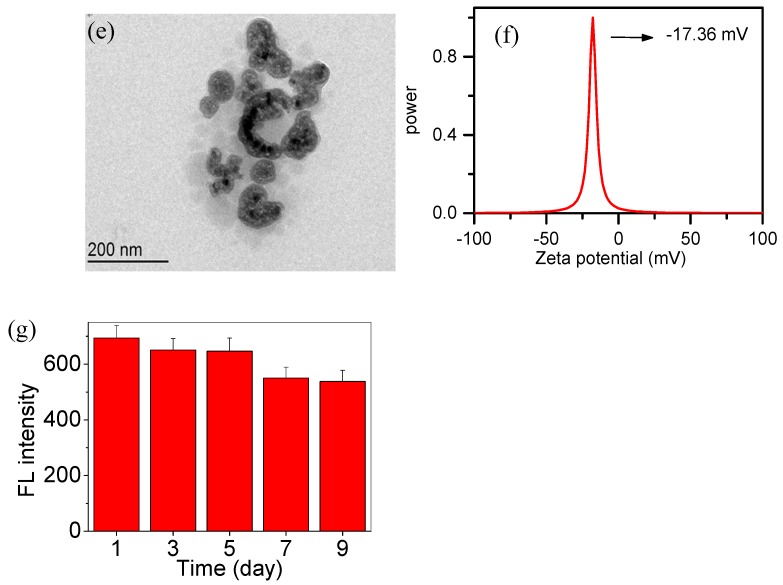
(**a**) Schematic illustration of AIE–Fe nanodots formation. (**b**) Dynamic Light Scattering (DLS) (**c**) absorption, (**d**) emission (λ_ex_ = 494 nm), (**e**) Transmission Electron Microscope (TEM), (**f**) Zeta potential spectrum and (**g**) fluorescence intensity of AIE–Fe nanodots for storing different time at room temperature (nanodots composition: 5 mg TPAS, 5 mg Fe_3_O_4_, 10 mg Pluronic F-127 in 10 mL water).

Such typical AIE character makes TPAS ideal for the fabrication of ultra-bright organic dots. An amphiphilic block copolymer, Pluronic F-127 was used as the matrix to encapsulate TPAS and Fe_3_O_4_ nanoparticles to form AIE–Fe hybrid nanodots (Figure 2a). Upon nanodots formation, the hydrophobic poly(propylene glycol) segments of the matrix intertwine with TPAS and Fe_3_O_4_ to form compact aggregates which makes up the core, while poly(ethylene glycol) chains render outside towards the water phase, stabilizing the resultant AIE–Fe hybrid nanodots and rendering them with excellent colloidal stability.

Fe_3_O_4_ nanoparticles exhibit broad featureless absorption in the visible domain due to the electronic transition of d-orbitals. TPAS showed two absorption peaks at 298 and 494 nm. The UV–vis absorption spectrum of AIE–Fe nanodots is the sum of spectra of two components (Figure 2c). More importantly, AIE–Fe nanodots showed stronger intense NIR emission at 700 nm (Figure 2d) than TPAS nanoaggregates in presence of 95% water, although Fe_3_O_4_ nanoparticles are reported used as a fluorescence quencher [19]. The TEM image in Figure 2e showed the size of the AIE–Fe nanodots were around 100 nm. We can also see thin layers on the surfaces and black dots on the core of AIE–Fe nanodots, suggesting that Pluronic F-127 was successfully enwrapped TPAS and Fe_3_O_4_ to yield as-synthesized NPs. The AIE–Fe nanodots show hydrodynamic diameters of 107 nm and zeta potentials of −17.36 mV (Figure 2f), which also confirm the successful fabrication of desired nanoparticles. The stability of AIE–Fe nanodots was measured by fluorescence intensity. It can be found that there were no obvious changes from 1 day to 9 days (Figure 2g).

**Figure 3:**
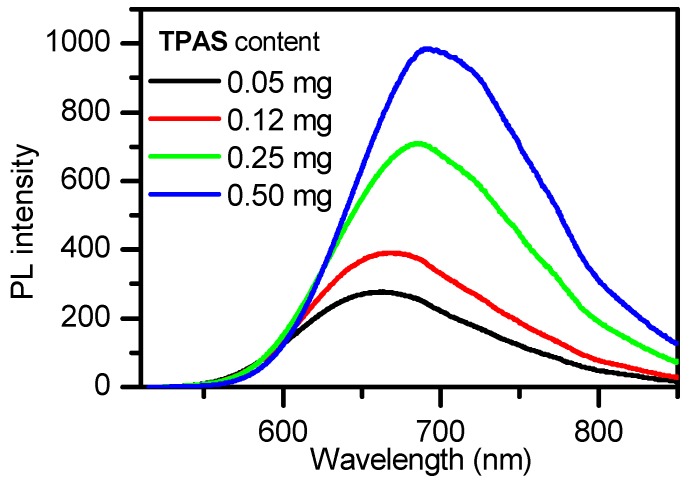
PL spectra of AIE–Fe nanodots containing different amount of TPAS (conditions: Fe_3_O_4_ 0.5 mg, Pluronic F127 10 mg, H_2_O 10 mL).

We further investigated the effect of Fe_3_O_4_ nanoparticles to on quantum yield (φ) of AIE–Fe nanodots (Table 1). It can be found that φ value slightly decreased with increasing amount of Fe_3_O_4_ nanoparticles, but it is still higher than that reported NIR-emission of nanodots. For example, Altinoğlu EI has reported a near-infrared emitting fluorophore-doped calcium phosphate nanoparticles whose quantum yield is just 4.9% [23]. The highest φ value was as high as 13.8% (entry 4). The DLS data revealed that hydrodynamic diameters ranged from 93.5 nm to 106.7 nm were present for these AIE–Fe nanodots. To study the correlation between concentration of TPAS and fluorescence intensity of AIE–Fe nanodots, different amount of (0.5 mg, 0.25 mg, 0.12 mg and 0.05 mg) was used to fabricate in AIE–Fe nanodots, whereas Fe_3_O_4_ NPs and Pluronic F-127 were kept constant. As shown in Figure 3, fluorescence intensity of AIE–Fe nanodots increased and the maximum emission red-shifted as the amount of the TPAS increased.

**Figure 4:**
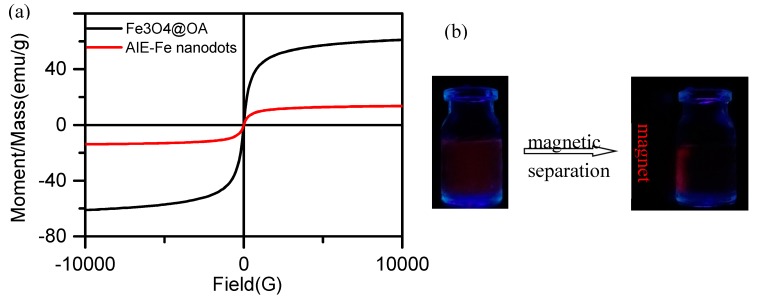
(**a**) Magnetization hysteresis loops of AIE–Fe nanodots and Fe_3_O_4_ nanoparticles. (**b**) The photograph of the AIE–Fe nanodots in an external magnetic field.

As shown in Figure 4, the magnetic hysteresis loops of AIE–Fe nanodots exhibited super paramagnetic behavior. The magnetic saturation value of the AIE–Fe nanodots was 14.2 emu g^−1^, which was lower than that of Fe_3_O_4_ nanoparticles, mainly caused by the F127 shell. The measured saturation magnetization suggested probability for improving the effect of MRI [21]. The inset in Figure 4 showed the photograph of the AIE–Fe nanodots in the presence of an external magnetic field, indicating the magnetic property of the nanoprobe was good enough for magnetic separation. The magnetic property was significant for the separation of analytes from the complicated detection system, thus reducing the influence of the interferences in the detection system.

**Figure 5:**
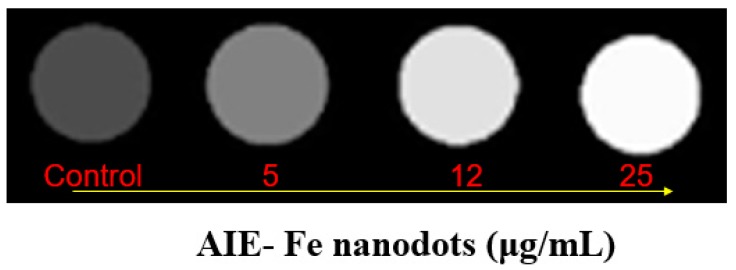
T1 weight images of AIE–Fe nanodots (with a 5 wt % TPAS loading).

As shown in Figure 5, the T1-weighted MRI contrast effect correlated with the concentration of the AIE–Fe nanodots. The filter paper in deionized water show poor contrast. The AIE–Fe nanodots displayed an enhancement in the T1-weighted MR signal with the increasing Fe concentration. This confirmed that the AIE–Fe nanodots could be utilized as promising T1 mode contrast agents for bioimaging.

**Figure 6:**
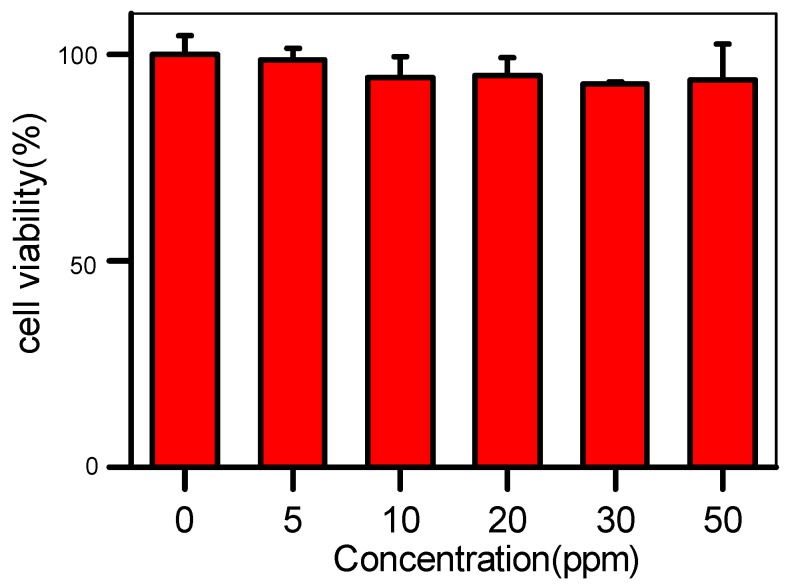
Cell viability of HeLa cells treated with different concentrations of AIE–Fe nanodots (0, 5, 10, 20, 30 and 50 ppm) for 24 h via MTT assay.

**Figure 7:**
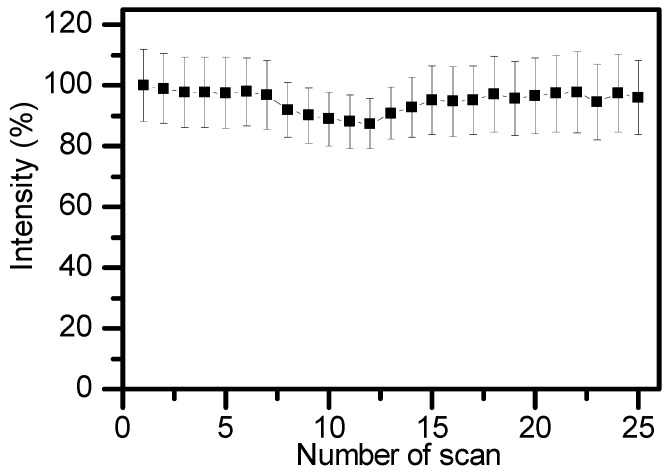
Photostability of AIE–Fe nanodots (10 ppm).

To study the in vitro cytotoxicity of AIE–Fe nanodots, HeLa cells were treated with different concentrations of AIE–Fe nanodots by a cell-counting MTT assay. As shown in Figure 6, the cell viability remains u to 90% after 24 h-incubation even with high concentration of AIE–Fe nanodots. In addition, AIE–Fe nanodots system exhibited high photostability. As shown in Figure 7, after 25 cycles confocal laser scanning its fluorescence signals remain almost constant. This might be due to the AIE nature of the probe, which is capable of maintaining highly emissive in the aggregation state [22].

**Figure 8:**
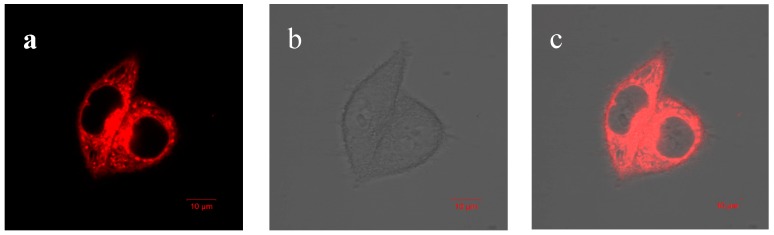
Confocal microscopy images of HeLa cells incubated with AIE–Fe nanodots (10 ppm) for 1 h: (**a**) in the red channel, (**b**) in the bright field, (**c**) merged image of (**a**) and (**b**). Scale bar:10 μm.

The low cytotoxicity and good photostability of AIE–Fe nanodots guarantees its uses in cells bioimaging. Then, the confocal fluorescent imaging was performed in HeLa cells with AIE–Fe nanodots (10 ppm) was used to stain the cells. As shown in Figure 8, HeLa cells were incubated with AIE–Fe nanodots for 24 h; obvious red emission was observed.

## 4. Conclusions

We designed and synthesized novel multifunctional AIE–Fe nanodots with spherical morphology, uniform small size and good stability in water. The nanodots showed excellent NIR luminescence at 700 nm owing to AIE-active TPAS and an excellent magnetic resonance effect due to the presence of magnetic Fe_3_O_4_. As a result of low toxicity and high photostability, AIE–Fe nanodots are suitable for multimodal imaging applications, such as fluorescence imaging and MR imaging.

## Figures and Tables

**Table 1:** The effect of Fe_3_O_4_ nanoparticles on quantum yield (φ) of AIE–Fe nanodots (conditions: TPAS 0.5 mg, Pluronic F127 10 mg, H_2_O 10 mL).

No.	Fe_3_O_4_ (mg)	Φ (%)	DLS Size (nm)
1	0.50	10.5	106.7
2	0.25	10.9	93.5
3	0.12	12.9	99.7
4	0.05	13.8	102.8

## Supplementary Materials

The following are available online at http://www.mdpi.com/2073-4360/11/2/220/s1, Figure S1, 1H NMR spectrum of 1a. Figure S2, 1H NMR spectrum of 1b. Figure S3, 1H NMR spectrum of TPAS. Figure S4, 13C NMR spectrum of TPAS. Figure S5, HRMS spectrum of TPAS.
