# Supplementary material for: Fabrication and Application of Dual-Modality Polymer Nanoparticles Based on an Aggregation-Induced Emission-Active Fluorescent Molecule and Magnetic Fe3O4
Source: Polymers (Basel). 2019 Jan 28;11(2):220. doi: 10.3390/polym11020220 (PMC6419270; doi:10.3390/polym11020220)
Supplement: Supplementary file 1 [file polymers-11-00220-s001.pdf]

Supporting information

Fabrication and Application of Dual-Modality Polymer Nanoparticles Based on an AIE-Active Fluorescent Molecule and Magnetic Fe_3O_4

Lingyun Wang [1,*], Meiying Huang [1], Hao Tang [1], Derong Cao [1] and Yu Zhao [2]

[1] School of Chemistry and Chemical Engineering, South China University of Technology, Guangzhou 510640, China; hmy_1015@126.com (M.H.); haotang@scut.edu.cn (H.T.); drcao@scut.edu.cn (D.C.)
[2] Shanghai Key Laboratory of Magnetic Resonance and Department of Physics, East China Normal University, 3663 North Zhongshan Road, Shanghai 200062, China; zhaoyu2013022063@163.com
* Correspondence: lingyun@scut.edu.cn; Tel. +86-20-87110245; Fax: +86-20-87110245

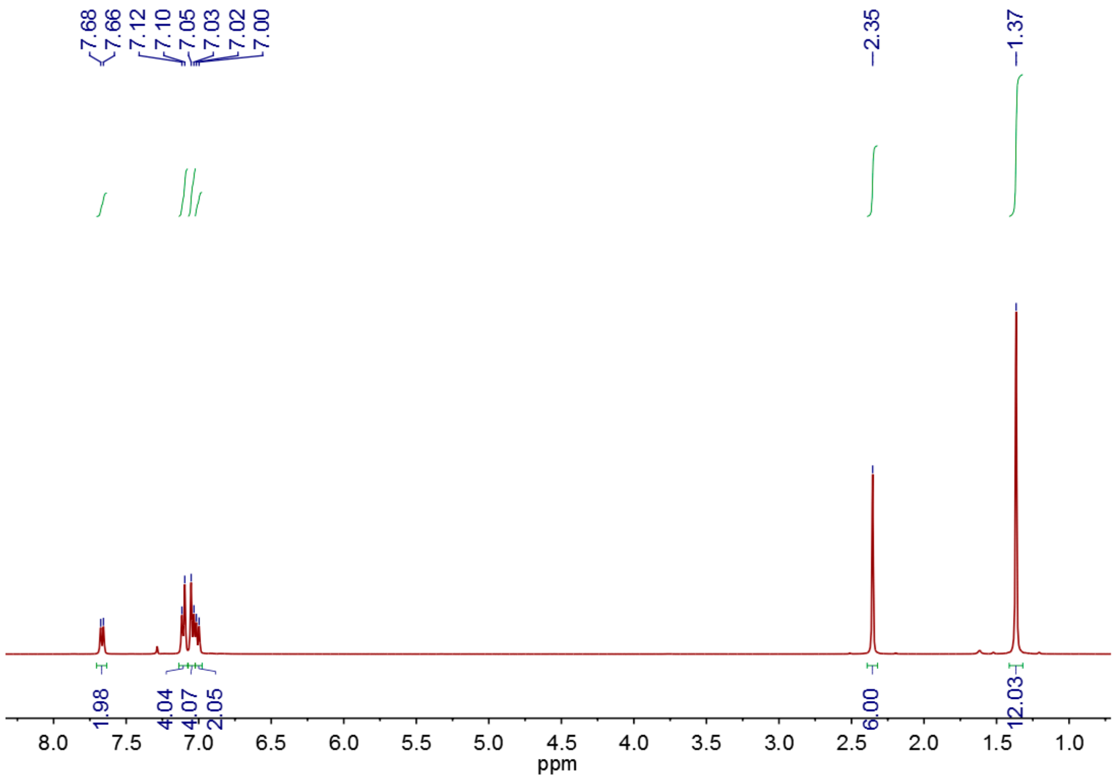

Figure S1. ^{1}H NMR spectrum of **1a**.

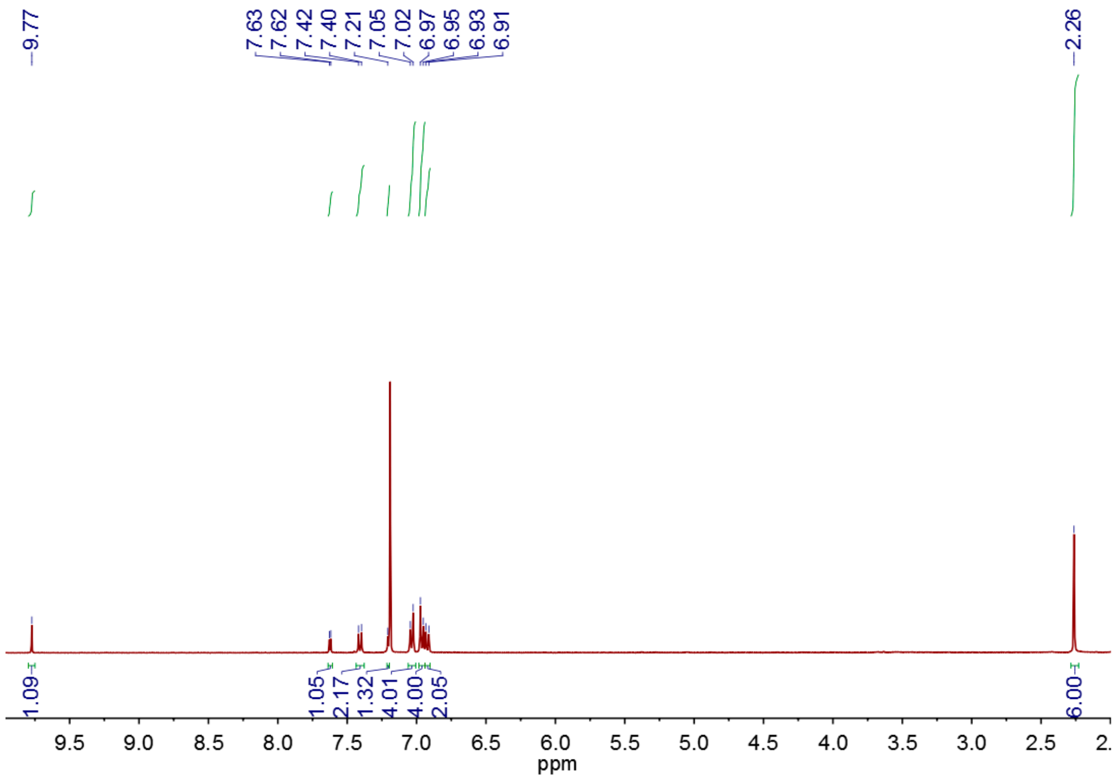

Figure S2. ^{1}H NMR spectrum of **1b**.

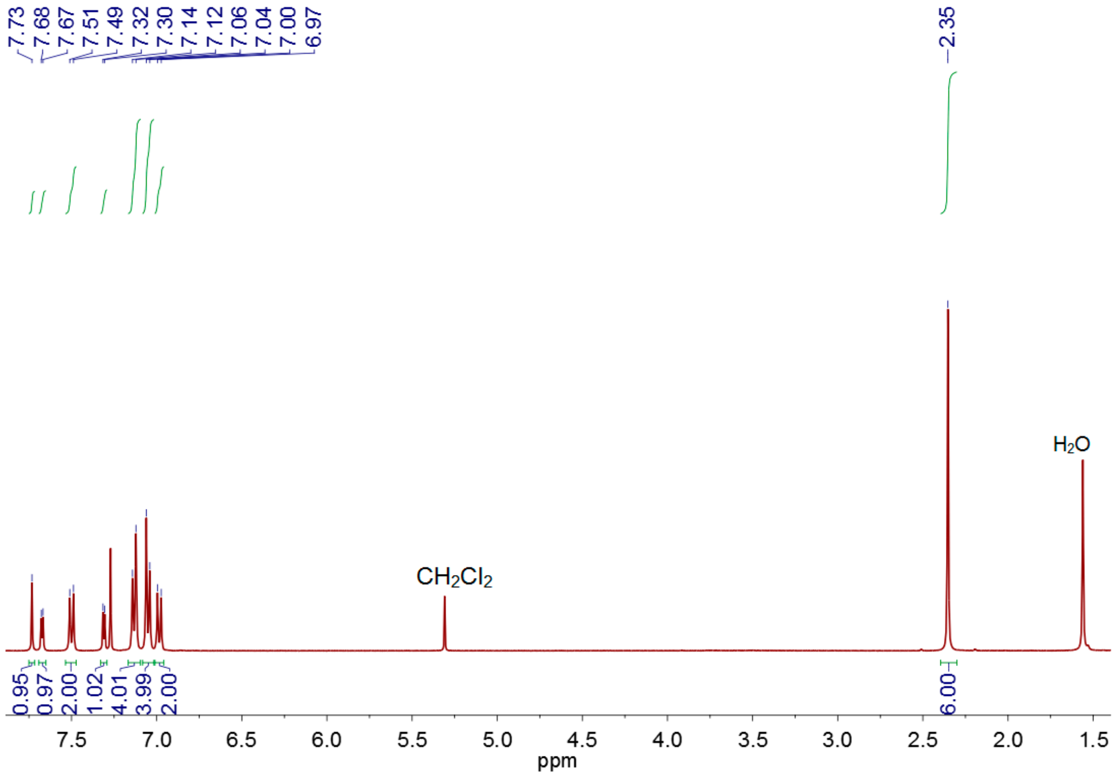

Figure S3. ^{1}H NMR spectrum of **TPAS**.

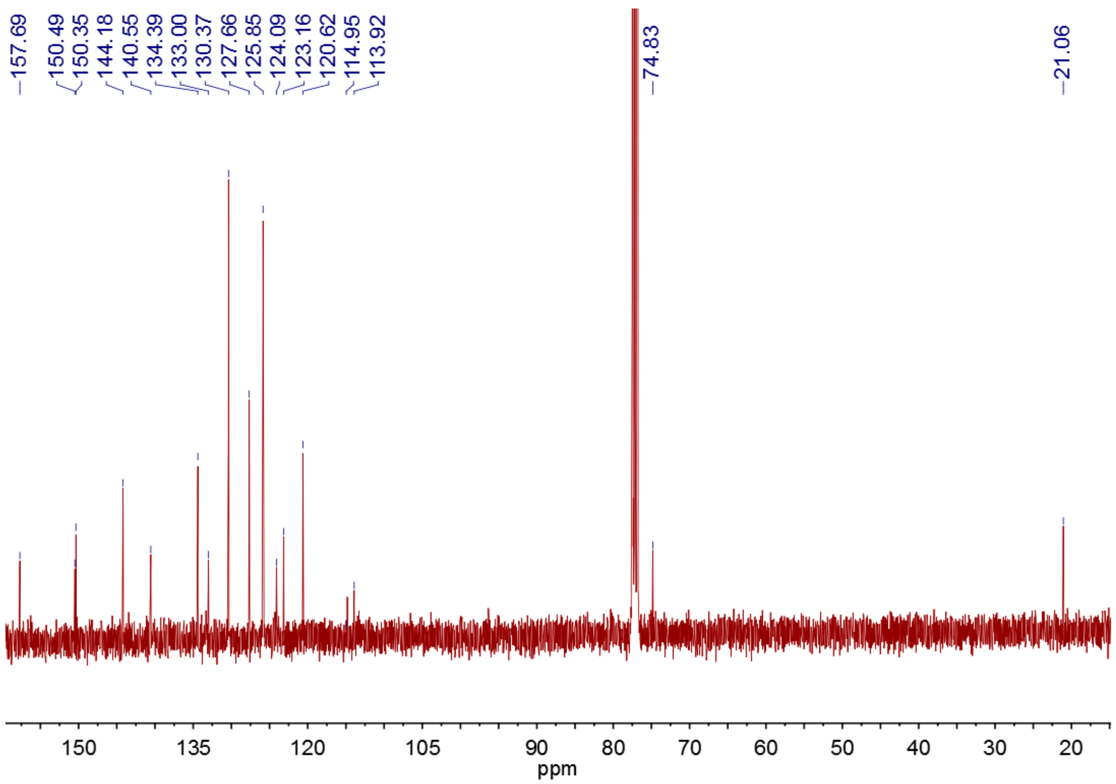

Figure S4. ^{13}C NMR spectrum of **TPAS**.

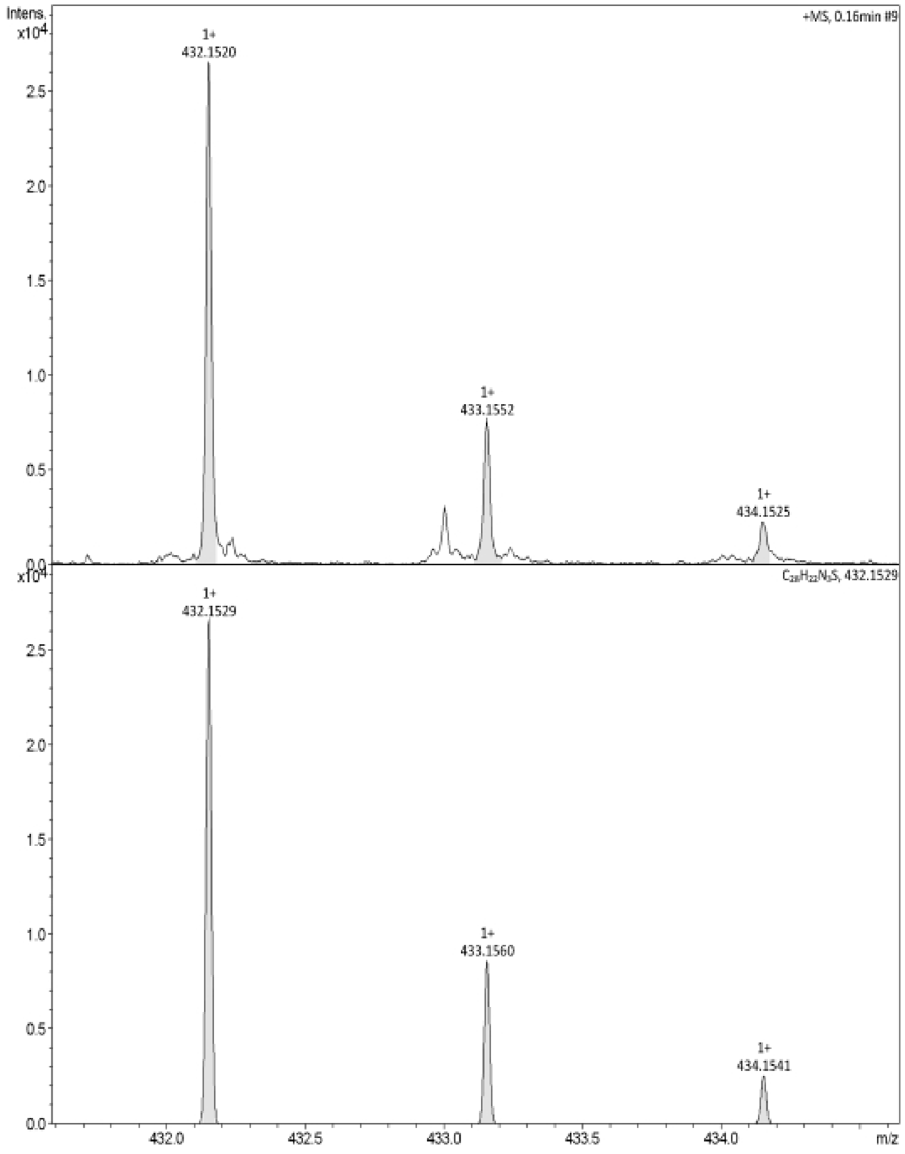

Figure S5. HRMS spectrum of **TPAS**.
